# Preservation of allograft bone using a glycerol solution: a compilation of original preclinical research

**DOI:** 10.1186/s40824-019-0154-1

**Published:** 2019-02-13

**Authors:** Brian Samsell, Davorka Softic, Xiaofei Qin, Julie McLean, Payal Sohoni, Katrina Gonzales, Mark A. Moore

**Affiliations:** LifeNet Health, 1864 Concert Drive, Virginia Beach, VA 23453 USA

**Keywords:** Glycerol, Tissue preservation, Allograft, Freeze-dried, Frozen, Preservon

## Abstract

**Background:**

Bone allografts are used in many orthopedic procedures to provide structural stability as well as an osteoconductive matrix for bone ingrowth and fusion. Traditionally, bone allografts have been preserved by either freezing or freeze-drying. Each of these preservation methods has some disadvantages: Frozen grafts require special shipping and storage conditions, and freeze-drying requires special lyophilization equipment and procedures that may impact biomechanical integrity. This report describes an alternate type of preservation using glycerol, which allows storage of fully-hydrated tissues at ambient temperature avoiding the potential complications from freeze-drying.

**Methods:**

In the in vitro three-point bend test, cortical bone was processed and frozen, freeze-dried, or treated with glycerol-based preservation (GBP). Load was applied to each graft at a rate of 2.71 mm/min. The flexural strain, flexural strength, and flexural modulus were then calculated. In the in vitro axial compression test, iliac crest wedges, fibular segments, and Cloward dowels were processed and either freeze-dried or GBP treated. The compressive strength of the grafts were tested at time zero and after real time aging of 1, 4, and 5 years. In the in vivo rat calvarial defect assessment, freeze-dried, frozen, and GBP bone implants were compared after being implanted into a critical sized defect. Samples underwent histological and biomechanical evaluation.

**Results:**

Bone grafts subjected to GBP were found to be at least biomechanically equivalent to frozen bone while also being significantly less brittle than freeze-dried bone. GBP-preserved bone demonstrated significantly greater compressive strength than freeze-dried at multiple time points. Preclinical research performed in calvaric defect models found that GBP-preserved bone had similar osteoconductivity and biocompatibility to frozen and freeze-dried samples.

**Conclusion:**

Preclinical research demonstrated that glycerol–preservation of bone yields a material that maintains biomechanical strength while eliminating the need for extensive rehydration or thaw periods if used clinically. Additionally, in vivo evidence suggests no negative impact of glycerol-preservation on the ability of bone grafts to successfully participate in new bone formation and fusion.

## Background

For over a century, bone allografts have been used by surgeons to restore structure and stability to patients with a variety of bone ailments. Allografts for human clinical use are tissues gifted from either living or deceased human donors. By contrast, autograft is bone often taken from an additional surgery site on the patient. While many surgeons still consider autograft the gold standard, allografts have gained popularity because they avoid the potential for donor site morbidity and pain [1] and varying quality. For the last several decades, the use of bone allografts has become increasingly common for clinical procedures including spinal fusion, high tibial osteotomies, dental implants, and limb salvage [2–4]. In fact, the number of bone allografts used in surgical procedures in the United States increased from an estimated 5000–10,000 in 1985 to 145,000 in 1996 [5]. For comparison, bone autografts were used in 247,000 of the 426,000 bone grafting procedures reported in 1996. The number of allograft procedures has continued to increase with over one million musculoskeletal allografts implanted annually in the United States alone [6]. Bone allografts have also become popular with surgeons because they are readily available in a variety of precise shapes and sizes. These preformed grafts can save valuable operating room time because the surgeon or operating room staff do not have to spend time shaping the bone as they would autograft. The use of allogeneic tissue may also benefit the patient by reducing time under anesthesia as well as donor site pain.

Traditional preservation methods for allografts include freezing or freeze-drying. Frozen grafts require special shipping and storage conditions as well as the need to thaw the graft before use. Freeze-dried grafts must be rehydrated before use, which may not be able to restore the bone’s native properties. A partially hydrated implant may be biomechanically compromised [7]. Additionally, immunogenic response has also been reported as a concern with freeze-dried allografts as this process may preserve the antigens in bone [8–10]. Collective concerns regarding immune response, pre-surgery preparation, alteration of biomechanical properties, shipping, and storage requirements prompted the development of an alternative to freezing and freeze-drying. A glycerol-based preservation (GBP) process was developed that exploits the properties of glycerol to protect tissue and keep it fully hydrated, avoiding freeze-drying and associated costs and tissue alterations and while still allowing for the convenience and reduced costs associated with shipping and storage at ambient temperatures compared to frozen or refrigerated tissue. Glycerol is a non-toxic, biodegradable liquid that the FDA classifies as ‘Generally Recognized as Safe’ (GRAS) [11]. It is a ubiquitous sweetener and preservative in over 1500 food, cosmetic, and pharmaceutical products. Furthermore, glycerol has been safely and effectively used as a carrier in demineralized bone matrices for spine fusion procedures for almost 2 decades [12–14] and is more recently being used to preserve decellularized dermis [15]. The GBP (*Preservon®, LifeNet Health, VA Beach, VA*) process utilizes glycerol to protect bone and soft allograft tissue [16]. As detailed in Crouch and Wolfinbarger Jr. [16], GBP provides a method of preserving bone and soft tissue grafts by replacing water molecules in the tissue with glycerol. Glycerol has a low molecular weight, which allows it to replace the water molecules by filling in available space within the tissue structure. By keeping the grafts moist, glycerol allows bone and dermis allografts to be stored at room temperature without drying out.

**Fig. 1:**
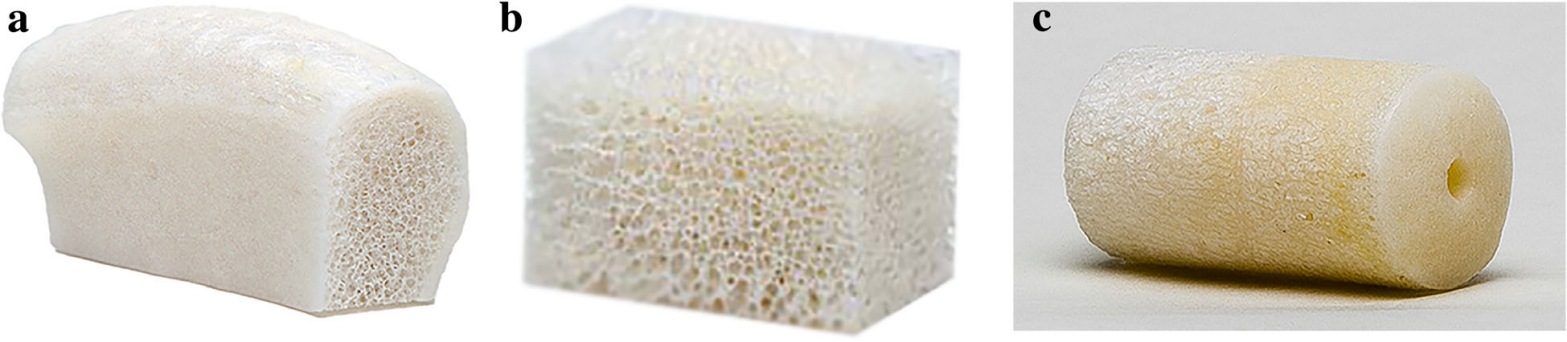
Examples of bone that have undergone glycerol-perseveration (GBP): **a** ilium strip with two cortical sides and a cancellous matrix, **b** cancellous bone block, and **c** cancellous bone dowel with thin cortical plate. Reproduced with permission from LifeNet Health

Other important characteristics of any preservant are to be non-toxic, compatible with the tissue, and to not alter the orientation of collagen fibers. Preservants can be introduced at several different points during processing, but typically occurs after tissue has been cleaned and disinfected. Bone tissue is initially cleaned with a solution of mild chemicals, detergents, and antibiotics [17]. This solution is induced to flow through the bone tissue using negative pressure, along with sonification, centrifugation, and peroxide exposure [18]. The induced flow cleans the bone by removing the bone marrow while also acting as a disinfection step. The tissue is then treated using a glycerol solution. An induced flow is used to distribute glycerol throughout the entire tissue Excess glycerol is then removed using a dry spin or by blotting. Finally, the tissue is packaged and a controlled low dose of gamma irradiation (< 20 kGy) is applied at ultra-low temperatures to provide a sterility assurance level (SAL) of 10^− 6^. The end result is terminally sterilized, glycerol-preserved bone tissue that can be stored at ambient temperature until use (Fig. 1). If intended for clinical use, the tissue may be rinsed briefly before use.

This report describes a compilation of original preclinical research evaluating the biomechanical and biochemical properties of GBP-treated bone allografts.

## Methods

### In vitro biomechanical studies

#### Three-point bend test

Cortical bone recovered from the femoral shaft of seven donors was procured and cut into 120 rectangular bars measuring 4 mm × 4 mm × 80 mm for biomechanical testing [19]. After undergoing a cleaning and disinfection protocol, the bars were divided into three different treatment groups. Grafts in control groups were either freeze-dried or frozen. Eighty of the one hundred-twenty rectangular bars were freeze-dried, packaged, gamma irradiated on dry ice with an absorbed dose of 13.3 to 17.0 kGy, and then stored at ambient temperature until testing. An additional 20 of the 120 rectangular bars were packaged, gamma irradiated on dry ice with the same absorbed dose, and stored in a -80 °C freezer until testing. The remaining 20 rectangular grafts were assigned to the experimental group and underwent the GBP process. For this analysis, the samples were treated with 77%-v:v glycerol using at least a 1:5 bone mass to solution volume ratio and were packaged, gamma irradiated on dry ice with the same absorbed dose, and stored at ambient temperature until testing. Prior to testing, the rectangular bars from the GBP group underwent a 30 s rinse consisting of ambient temperature 0.9% isotonic saline. The frozen bars received a 2 min rinse, and freeze-dried bars had 1 of 4 different rinses ranging from 30 s to 60 min. The American Society for Testing and Materials (ASTM) D790–97 Procedure A test method was used from Instron Bluehill software. Load was applied to each graft at a rate of 2.71 mm/min. The flexural strain (Table 1), flexural strength (Table 2), and flexural modulus were then calculated. Flexural strain was determined by normalizing the level of deflection during three-point bend testing using specimen geometry. Flexural strength was derived by normalizing maximum applied force using specimen geometry. Flexural modulus was defined as the maximum slope of the linear region of the flexural strength and flexural strain curve. A one way analysis of variance (ANOVA) with a confidence interval of 95% was used to compare biomechanical properties among groups. A Tukey’s post-hoc test was performed for differences found in the ANOVA analysis.

**Table 1:** Equation used to calculate flexural strain

Є_f_ = 6Dd / L^2^	
Є_f_ = strain in the outer surface, mm/mm	
D = maximum deflection of the center of the beam, mm	
L = support span, mm	
d = depth, mm

**Table 2:** Equation used to calculate flexural strength

σ_f_ = 3YL / 2bd^2^	
σ = stress in the outer fibers at midpoint, MPa	
Y = yield point, N	
L = support span, mm	
b = width of beam tested, mm	
d = depth of beam tested, mm	

#### Axial compression testing

Iliac crest wedges (0.9 cm), fibular segments (2.0 cm), and Cloward dowels (1.4 cm) were selected as bone grafts for testing. Following a cleaning and disinfection protocol, grafts were either GBP treated or freeze-dried, packaged, stored in PETG trays, and then, for testing purposes, gamma irradiated on dry ice with an elevated absorbed dose of 25 kGy. Prior to testing, freeze-dried grafts were rehydrated in ambient saline for a minimum of 60 min for load bearing grafts. GBP grafts were rinsed in ambient saline for a minimum of 5 min. Cross-sectional area was measured for all the grafts tested. Iliac crest wedges were measured using a planimeter due to their irregular shape. Iliac crest wedges were measured in triplicate and then averaged because of their different top and bottom surfaces. Fibular segments were also measured using a planimeter. Both surfaces were measured in triplicate and averaged for the same reasons as above. Total cross-sectional area was calculated by (Area outside – Area inside). To determine the cross-sectional area of the Cloward dowels, the diameter (D) and length (L) of each specimen was measured in triplicate and averaged. Cross-sectional area was then calculated using the formula (πDL)/4.The Traditional Bone Grafts – Axial Compression test method was used from Instron Bluehill software. Iliac crest wedges and fibular segments were tested on standard platens, whereas the Cloward dowels were tested on custom milled compression platens. These platens allowed the bio-implant to lay in rounded grooves that match the shape of the bio-implant to ensure that the load is applied evenly across the length of the dowel. Pertinent information, including cross-sectional area and gauge length, was entered into the software. Each sample was loaded to failure at a rate of 35 mm/min using an Instron mechanical testing machine that applied compressive load.

The compressive strength of iliac crest wedges, fibular segments, and Cloward dowels were tested at time zero and after real time aging of 1, 4, and 5 years. The compressive strength of test groups at each time group was compared using a t-test at a 95% confidence interval. The results from the real time five-year time point were then evaluated against results from the baseline time point, real time one-year time point, and real time four-year time point using a one way analysis of variance (ANOVA) with a confident interval of 95%. If differences were found, then the analysis was followed by a post hoc test, Turkey’s method modified for unequal sample sizes, in order to determine which groups were statistically different from each other.

### In vivo rat Calvarial defect assessment

Freeze-dried, frozen, and GBP bone implants were compared using a rat calvarial defect model [20] in skeletally mature, male Wistar rats. This study was performed in accordance with guidelines and regulations set forth by the Animal Welfare Act and the University of Maryland Institutional Animal Care and Use Committee (IACUC), and received approval by the IACUC (reference number 04–09-04). In the calvarial defect model, part of the rat’s skull is removed to create a critical size defect, meaning a wound that could not naturally heal on its own [21]. A bone implant of smaller size is implanted into the defect site to test both osteoconductivity and biocompatibility of the implant. An autologous bone implant derived from the defect creation in each rat was used as a control. In the first set of implants, the rats were bilaterally grafted with 4-mm bone discs into a 5-mm circular defect (Table 3). Human cortical and cancellous bone discs from the same donor were disinfected as described above and then differentially preserved (*n* = 6 for frozen, *n* = 6 for freeze-dried, *n* = 12 for GBP). Control grafts (*n* = 4) consisted of autologous bone discs derived during the defect creation. Animals were euthanized at one week, and samples were excised, stained with H&E, and evaluated histologically for inflammation. A trained histologist and board-certified pathologist (Michael Bergevin, MD, Medical Director, LifeNet Health) performed a blinded review. In the second set of animals, the same procedure was followed as above, but the grafts were explanted after six weeks rather than after one week (Table 3). Cortical and cancellous bone samples were excised and tested for push-out strength using a three-point bend testing, which provides a mechanical measurement of the comparative degree of healing between graft materials. The third and final set of animals were grafted with 5 mm diameter cortical and cancellous bone discs from the three different preservation groups in 5 mm defects to provide a tighter press-fit (Table 3). After six weeks, defect closure was assessed via histology and radiography. Radiography images were digitized for densiometric analysis. Density was measured in pixels and was calculated as the difference between 4.5 mm from the center of the defect and 5.5 mm from the center of the defect, thus isolating the region of interest as a 1 mm band encompassing the defect margin (Table 4).

**Table 3:** Rat Calvarial Defect Study Design

	*n* (rats)	Disc size (mm)	Defect size (mm)	Time point (weeks)
First group				
Frozen	6	4	5	1
Freeze-dried	6	4	5	1
GBP^a^	12	4	5	1
Autologous control	4	4	5	1
Second group				
Frozen	6	4	5	6
Freeze-dried	6	4	5	6
GBP	12	4	5	6
Autologous control	4	4	5	6
Third group				
Frozen	6	5	5	6
Freeze-dried	6	5	5	6
GBP	12	5	5	6
Autologous control	4	5	5	6

^a^Glycerol-based preservation

**Table 4:** Densiometric analysis

Bone type	Treatment	Average Pixel Density × 10^3^ (± SD)
Cortical Samples	Frozen	297.3 ± 14.6
	Freeze-dried	313.4 ± 27.8
	GBP	304.8 ± 25.1
Cancellous Samples	Frozen	272.7 ± 14.9
	Freeze-dried	280.8 ± 31.7
	GBP	294.7 ± 24.9

## Results

### In vitro biomechanical studies

#### Three-point bend test

**Fig. 2:**
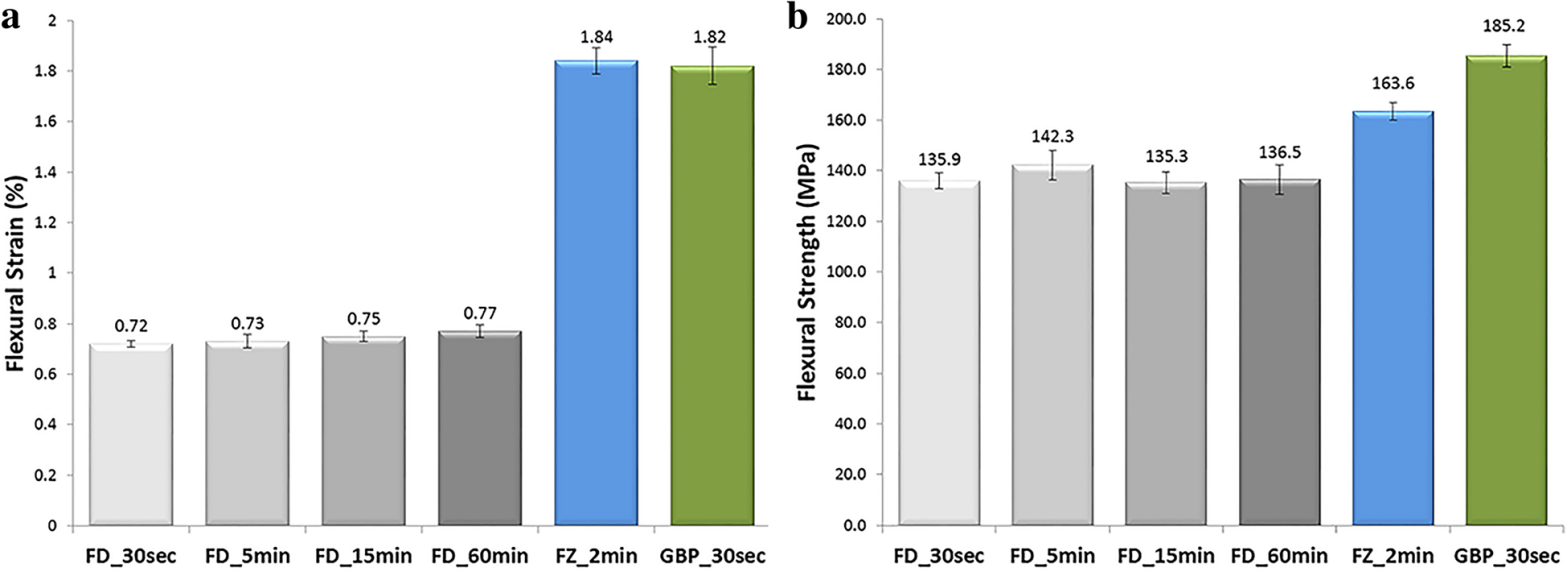
Flexural strain and flexural strength of the three preservation groups as evaluated using a three-point bend test**. a** Average maximum flexural strain. The glycerol-preservation (GBP) and frozen groups were not significantly different, but both demonstrated significantly greater average maximum flexural strain compared to the freeze-dried group. **b** Average maximum flexural strength. The GBP, frozen, and freeze-dried groups were each significantly different from one another

Both the GBP and frozen groups demonstrated significantly greater average maximum flexural strain compared to the freeze-dried group (Fig. 2a). There were no significant differences between the frozen and GBP groups, or between the individual subgroups with different rinse times in the freeze-dried arm. The GBP group demonstrated the greatest average flexural strength, and this was statistically greater than both the frozen and freeze-dried groups (Fig. 2b). The frozen group showed significantly greater average flexural strength than the freeze-dried group, which displayed the lowest. There was no difference in average flexural strength among the different subgroups with different rinse times in the freeze-dried group, which suggested a generally non-reversible alteration of the bone matrix upon freeze-drying. Both the GBP and frozen groups demonstrated significantly lower average flexural modulus than the freeze-dried group.

#### Axial compression testing

**Fig. 3:**
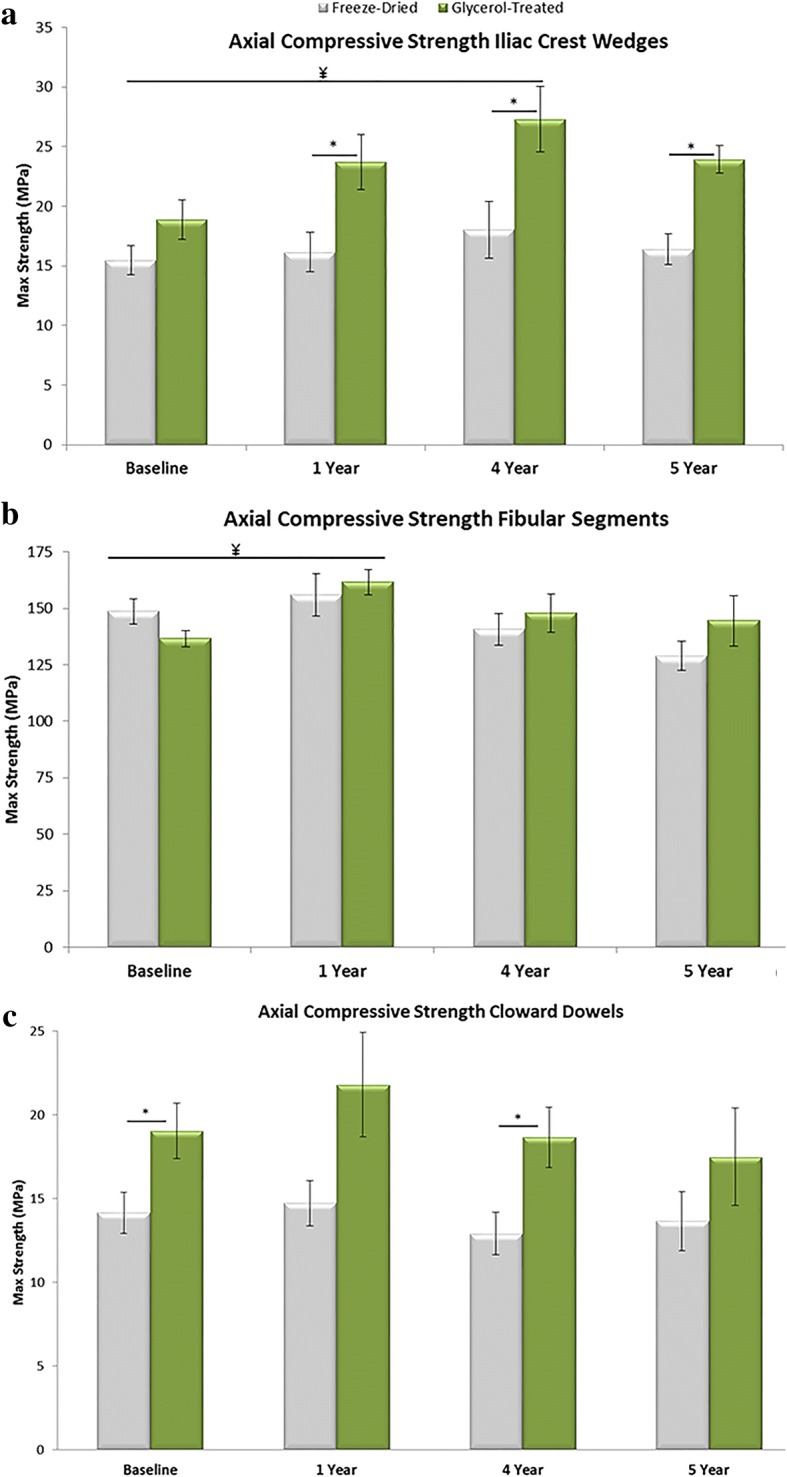
Axial compressive strength of different bone grafts treated with glycerol-preservation (GBP) or freeze-drying **a)** Axial compressive strength of iliac crest wedges. **b** Axial compressive strength of fibular segments. **c** Axial compressive strength of Cloward dowels. ¥ statistical significance between GBP at a given time point and both baseline groups (*p* < 0.05). *statistical significance between GBP and freeze-dried groups at given time point (*p* < 0.05)

At the five-year real time aging point, the compressive strength of GBP iliac crest wedges was significantly stronger than the freeze-dried group (*p* < 0.01) (Fig. 3a). The effect of real-time aging did not significantly affect either group. At the five-year real time aging point, the compressive strength of GBP fibular segments was similar to the freeze-dried group (*p* = 0.24) (Fig. 3b). The effect of real time aging on the compressive strength was not significant for fibular segments using either preservation method (freeze-dried group (*p* = 0.27); GBP group (*p* = 0.15). At the five-year real time aging point, the compressive strength of GBP Cloward dowels was similar to the freeze-dried group (*p* = 0.27) (Fig. 3c); however, GPB demonstrated significantly greater strength than freeze-dried at baseline and at 4 years. The effect of real time aging on the compressive strength did not cause significant decreases in strength for Cloward dowels using either preservation method (freeze-dried group (*p* = 0.83); GBP group (*p* = 0.65)).

### In vivo rat Calvarial defect assessment

**Fig. 4:**
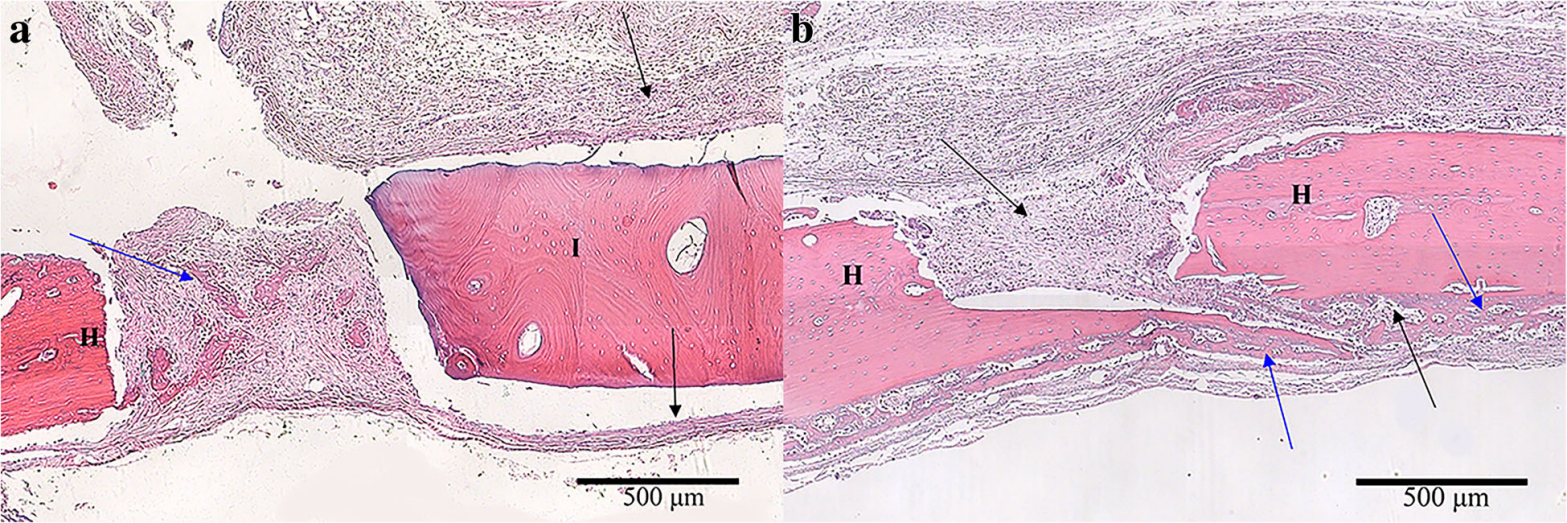
**a** Glycerol-preserved (GBP) cortical bone disc in calvaric defect model at 1 week. The scale is 500 μm. Black arrows indicate the connective tissue surrounding the implant, which resembles normal fibroblast-like cell organization. The blue arrow emphasizes new bone development in the host-implant junction (blue arrow). **b** Autogenous bone disc in calvaric defect model at 1 week. The scale is 500 μm. Black arrows indicate connective tissue infiltration, and blue arrows mark new bone development initiating the bone bridge formation. *I = Implant bone. H = Host bone

**Fig. 5:**
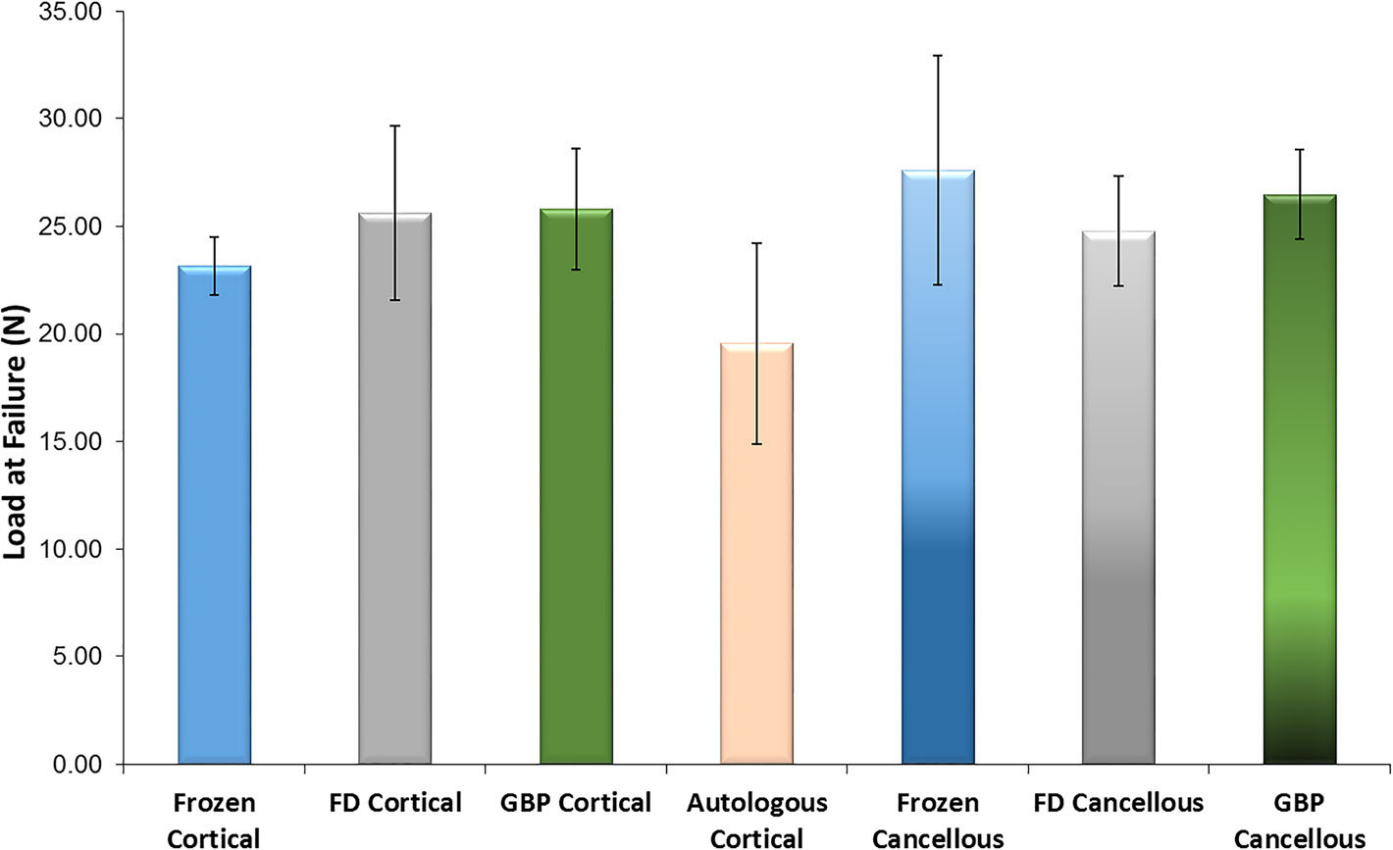
Average load at failure for rat calvarial defect – deformation analysis. Glycerol-preservation (GBP) groups were not statistically different than freeze-dried groups for any parameter (*p* > 0.05)

**Fig. 6:**
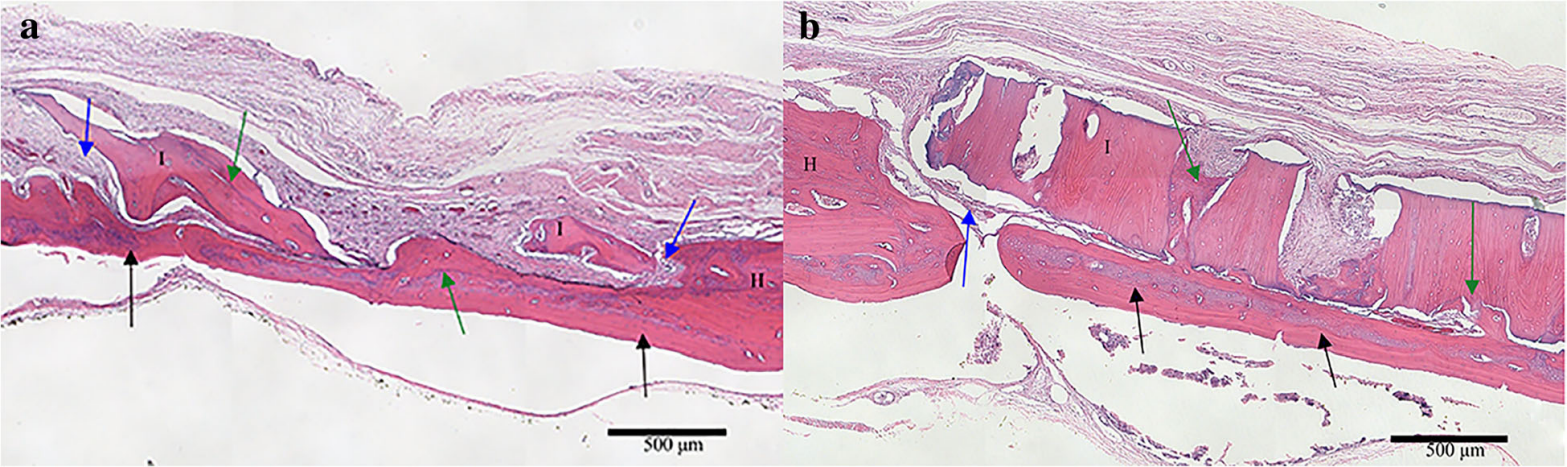
**a** Glycerol-preserved (GBP) cancellous bone in a tight-fit rat calvarial defect. The scale is 500 μm. **b** Freeze-dried cortical bone in a tight-fit rat calvarial defect at 6 weeks. The scale is 500 μm. Black arrows mark complete bone bridge formation. Blue arrows indicate soft tissue infiltrate. Green arrows mark osteointegration. H = Host bone. I = Implant bone

**Fig. 7:**
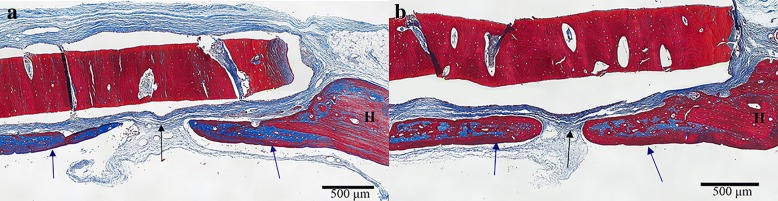
**a** Glycerol-preserved (GBP) cortical bone disc in calvaric defect model at 6 weeks. The scale is 500 μm. **b** Frozen bone disc in calvaric defect model at 6 weeks. The scale is 500 μm**.** For both images, black arrows indicate connective tissue infiltration and blue arrows mark new bone development initiating the bone bridge formation

In the first set of animals, the blinded reviewer did not observe differences in cellular response between any of the groups. Although some moderate leukocyte infiltration was observed, this was consistent with anticipated early post-operative inflammatory changes and occurred in both experimental (Fig. 4a) and autograft samples (Fig. 4b). In the second set of animals, neither cortical nor cancellous GBP groups were statistically different than freeze-dried groups for average load at failure (Fig. 5) or average peak load. Cortical GBP bone disc values were similar to the frozen group; however, the cancellous GBP group demonstrated statistically higher for peak load (*p* = 0.04) than the frozen cancellous bone discs, though the impact of significance is questionable due to the small sample sizes. In the third set of animals, densiometric analysis revealed no significant differences in density between GBP, freeze-dried, or frozen preservation methods for cortical and cancellous samples (*p* > 0.05) indicating similar density of bone bridge formation. Histologic findings consistent with areas of osteointegration were evident in specimens of all treatment groups (Fig. 6). Bone discs acted as a scaffold allowing the formation of a bony bridge originating from the margins of the defect site subjacent to the disc. In two defects, graft material migrated laterally and superiorly over the parietal bone; these osteotomy defects did not heal and showed no evidence of bone bridging in the time course of the study. The autogenous bone discs demonstrated gap closure in some instances and, in others, showed connective tissue remaining in the juncture between the defect and the disc. Additionally, GBP and frozen cortical bone samples stained with Masson’s trichrome demonstrated almost complete bone formation (Fig. 7). A blinded review of the six week photomicrographs by an independent orthopedic surgeon revealed no significant differences in bone formation between GBP-treated and untreated bone discs. The reviewer also noted the strong formation of the bone bridge on the dural side of the implant.

## Discussion

These biomechanical studies have demonstrated that GBP-treated bone allografts have at least equivalent strength as frozen or lyophilized (freeze-dried) bone allografts. Both the GBP and frozen groups demonstrated significantly lower average flexural modulus than the freeze-dried group. In general, a lower flexural modulus of elasticity would be consistent with less brittle materials. There were also significant differences observed between the shortest and longest rinse time subgroups within the freeze-dried group. This suggests a longer rinse makes the material slightly less brittle, but was still significantly more brittle than non-freeze-dried test groups. The axial compressive strength of GBP-treated and freeze-dried cortical (iliac crest wedges), cancellous (fibular shaft segments), and cortical-cancellous (Cloward dowels) allograft bone were tested at baseline, one year, four years, and five years. GBP-preserved bone demonstrated significantly greater compressive strength than freeze-dried at multiple time points. These results demonstrate that GBP-treated bone is not weaker than freeze-dried bone and retains properties over time.

The one-week GBP-treated bone discs using the rat calvarial defect model demonstrated histologically equivalent soft tissue responses as the frozen and freeze-dried samples. These responses were consistent with early post-operative changes and demonstrate the biocompatibility of GBP-treated bone. Following six weeks in vivo, the GBP-treated and untreated bone discs demonstrated equivalent osteoconductivity as assessed by radiography and biomechanical testing. Additionally, the independent, blinded histological review of the explanted samples revealed no observable differences between GBP and frozen or freeze-dried bone. These experiments confirmed that GBP-treated human bone implants were equivalent to frozen and freeze-dried implants in regards to biocompatibility and osteoconductivity, which enables new bone formation.

To date, there have been two published clinical evaluations that confirmed the safety and efficacy of GBP-treated bone allografts in direct comparisons with either freeze-dried or frozen bone allografts (Table 5) [22, 23]. All allografts underwent treatments and low dose gamma irradiation applied at ultra-low temperatures, as described above. Graham, Samsell [22] compared GBP -treated Cloward dowel bone allografts to freeze-dried Cloward dowels in a prospective, randomized controlled trial composed of 106 subjects undergoing anterior cervical discectomy and fusion (ACDF). ACDF is used to alleviate symptoms of cervical spondylosis, radiculopathy, or myelopathy using structural autograft or allograft to restore disc height and to initiate fusion. This study randomly assigned 53 subjects (113 levels of surgery) to the GBP-treated group (referred to glycerol-preserved in this study) and 53 subjects (114 levels of surgery) to the freeze-dried group. Blinded investigators assessed subsidence at 3 and 6 months, and fusion at 6 months using radiography. Average subsidence was slightly greater in the freeze-dried group at both time points, but this difference was not significant. Both arms demonstrated > 95% fusion, with one patient in the GBP group showing persistent movement upon flexion and extension and one patient in the freeze-dried exhibiting pseudofusion. There were no significant differences between the two groups in terms of clinical outcomes or adverse events, demonstrating that the GBP-treated allografts were safe and effective, while allowing for potential significantly shorter preparation times by being stored fully hydrated at room temperature. The authors also noted that GBP-treated Cloward dowels demonstrated an equivalent or superior fusion rate as cervical total disc replacement, per literature comparisons, while costing less than half the amount in materials, per cervical motion segment.

**Table 5:** Summary of studies using glycerol-preserved (GBP) bone allograft for spine fusion

Study	Graft Type	Preservation	Patient #	Levels	3 Month Fusion	6 Month Fusion	12 Month Fusion	3 Month Avg^b^ Subsidence	6 Month Avg^b^ Subsidence
Graham [22]	Cloward Dowel	Glycerol-Preserved	53 (113 levels)	1–4	–	> 95%	–	2.09	2.12
		Freeze-Dried	53 (114 levels)	1–4	–	> 95%	–	2.73	2.83
Rodway [23]	CPBA^a^	Glycerol-Preserved	28 (64 Levels)	1–4	45.3% (29 Patients)	–	100%	–	–
		Frozen	37 (70 Levels)	1–4	41.4% (38 Patients)	–	100%	–	–

^a^Composite Pinned Bone Allograft

^b^Calculated by multiplying average level subsidence by number of cases for that level, then taking the sum for all levels and dividing by total number of patients

**Fig. 8:**
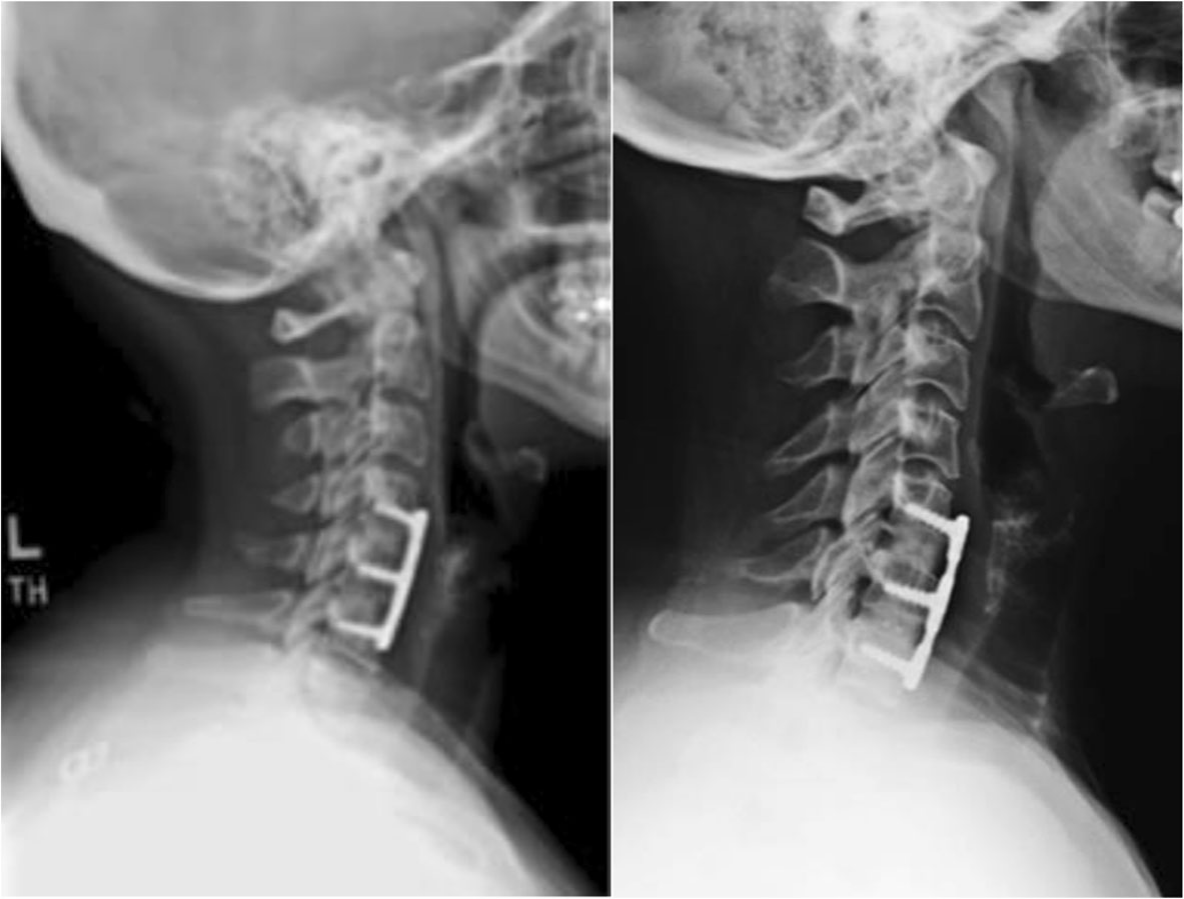
Six months postoperative X-ray images showing fusion for 2-level ACDF treated using frozen allografts (left) and glycerol-preserved (GBP) allografts (right). Reproduced from Rodway and Gander [23] under the Creative Commons Attribution License, which permits unrestricted use, distribution, and reproduction in any medium, provided the original work is properly cited

Additionally, a retrospective clinical study also found similar results in ACDF surgery for 28 subjects (64 levels) that received GBP-treated composite pinned bone allografts and 37 subjects (70 levels) that received frozen composite pinned bone allografts [23] (Fig. 8). After a one year follow-up, no significant differences were observed between the two groups with 100% rates of fusion and no reports of allograft-related complications in either treatment arm. The patient groups also experienced similar fusion rates early in the study at 3 months, indicating that clinical outcomes are similar beginning early in the post-operative time period. The authors concluded that GBP -treated grafts were found to be as safe and effective as frozen allografts.

## Conclusions

Preclinical research demonstrated that glycerol preservation of bone yields a material that maintains biomechanical strength while eliminating the need for extensive rehydration or thaw periods if used clinically. Additionally, in vivo evidence suggests no negative impact of glycerol-preservation on the ability of bone grafts to successfully participate in new bone formation and fusion.

## References

[1] McGuire DA, Hendricks SD (2009). Allograft tissue in ACL reconstruction. Sports Med Arthrosc.

[2] Buser Z, Brodke DS, Youssef JA, Meisel HJ, Myhre SL, Hashimoto R (2016). Synthetic bone graft versus autograft or allograft for spinal fusion: a systematic review. J Neurosurg Spine.

[3] Han JH, Kim HJ, Song JG, Yang JH, Bhandare NN, Fernandez AR (2015). Is bone grafting necessary in opening wedge high Tibial osteotomy? A meta-analysis of radiological outcomes. Knee Surg Relat Res.

[4] Monje A, Pikos MA, Chan HL, Suarez F, Gargallo-Albiol J, Hernandez-Alfaro F (2014). On the feasibility of utilizing allogeneic bone blocks for atrophic maxillary augmentation. Biomed Res Int.

[5] Boyce T, Edwards J, Scarborough N (1999). Allograft bone: the influence of processing on safety and performance. Orthop Clin N Am.

[6] American Association of Tissue Banks (AATB) (2007). American Association of Tissue Banks (AATB) Annual Survey of Accredited Tissue Banks in the United States.

[7] Bottino MC, Jose MV, Thomas V, Dean DR, Janowski GM (2009). Freeze-dried acellular dermal matrix graft: effects of rehydration on physical, chemical, and mechanical properties. Dent Mater.

[8] Pinkowski JL, Reiman PR, Chen SL (1989). Human lymphocyte reaction to freeze-dried allograft and xenograft ligamentous tissue. Am J Sports Med.

[9] Burchardt H, Glowczewskie F, Miller G. Freeze-dried segmental fibular allografts in azathioprine-treated dogs. Clin Orthop Relat Res. 1987;(218):259–67. 3568488

[10] Burchardt H, Jones H, Glowczewskie F, Rudner C, Enneking WF (1978). Freeze-dried allogeneic segmental cortical-bone grafts in dogs. J Bone Joint Surg Am.

[11] Food and Drug Administration. Title 21 § 182.1320. Glycerin. 2016.

[12] Cammisa FP, Lowery G, Garfin SR, Geisler FH, Klara PM, McGuire RA (2004). Two-year fusion rate equivalency between Grafton DBM gel and autograft in posterolateral spine fusion: a prospective controlled trial employing a side-by-side comparison in the same patient. Spine.

[13] Kang J, An H, Hilibrand A, Yoon ST, Kavanagh E, Boden S (2012). Grafton and local bone have comparable outcomes to iliac crest bone in instrumented single-level lumbar fusions. Spine (Phila Pa 1976).

[14] Thalgott JS, Fogarty ME, Giuffre JM, Christenson SD, Epstein AK, Aprill C (2009). A prospective, randomized, blinded, single-site study to evaluate the clinical and radiographic differences between frozen and freeze-dried allograft when used as part of a circumferential anterior lumbar interbody fusion procedure. Spine (Phila Pa 1976).

[15] Moore MA, Samsell B, Wallis G, Triplett S, Chen S, Jones AL (2015). Decellularization of human dermis using non-denaturing anionic detergent and endonuclease: a review. Cell Tissue Bank.

[16] Crouch K, Wolfinbarger Jr L, Inventors; LifeNet health, assignee. Plasticized bone grafts and methods of making and using same. United States patent US20100185284 A1. 2010.

[17] Wolfinbarger Jr L, Inventor; LifeNet health, assignee. Composition for cleaning bones. United States patent US5977034 a 1996.

[18] Wolfinbarger Jr L, Inventor; Lifenet Research Foundation, assignee. Process and composition for cleaning soft tissue grafts optionally attached to bone and soft tissue and bone grafts produced thereby. United States patent US6024735 a. 2000.

[19] Sohoni P, Morris AR, Balsly CR, Cotter AT, Sander TW (2011). The effects of a new preservation method on the biomechanics and shelf life of allograft bone. Annual meeting of the orthopedic research society.

[20] Spicer PP, Kretlow JD, Young S, Jansen JA, Kasper FK, Mikos AG (2012). Evaluation of bone regeneration using the rat critical size calvarial defect. Nat Protoc.

[21] Schmitz JP, Hollinger JO. The critical size defect as an experimental model for craniomandibulofacial nonunions. Clin Orthop Relat Res. 1986;205:299–308. 3084153

[22] Graham RS, Samsell BJ, Proffer A, Moore MA, Vega RA, Stary JM (2015). Evaluation of glycerol-preserved bone allografts in cervical spine fusion: a prospective, randomized controlled trial. J Neurosurg Spine..

[23] Rodway I, Gander J (2014). Comparison of fusion rates between glycerol-preserved and frozen composite allografts in cervical fusion. Int Sch Res Notices.

